# A new species of *Bothropolys* Wood, 1862 (Lithobiomorpha, Lithobiidae) from south-western China

**DOI:** 10.3897/BDJ.13.e149393

**Published:** 2025-03-27

**Authors:** Shiyi Gu, Sujian Pei, Huiqin Ma, Zixuan Zhang, Keying Zheng, Siqi Zhao, Zihan Zhang

**Affiliations:** 1 School of Life Sciences, Hengshui University, Hengshui, China School of Life Sciences, Hengshui University Hengshui China; 2 Hebei Key Laboratory of Wetland Ecology and Conservation, Hengshui, China Hebei Key Laboratory of Wetland Ecology and Conservation Hengshui China; 3 State Key Laboratory for Quality Ensurance and Sustainable Use of Dao-di Herbs, Beijing, China State Key Laboratory for Quality Ensurance and Sustainable Use of Dao-di Herbs Beijing China

**Keywords:** Chilopoda, centipede, *Bothropolysbiserialis* sp. nov., taxonomy, Chongqing Municipality

## Abstract

**Background:**

The genus *Bothropolys* is mostly known from North America and East Asia. So far eleven species of *Bothropolys* are reported in China.

**New information:**

A new lithobiid species, *Bothropolysbiserialis* sp. nov., is described and illustrated from the Yintiaoling National Nature Reserve in Wuxi County, Chongqing Municipality, south-western China. The new species is primarily compared with *B.yoshidai* Takakuwa, 1939, from the Hubei and Fujian Provinces, China, as well as Japan and North Korea and *B.curvatus* Takakuwa, 1939, from the Yunnan Province, China, as well as Japan and *B.rugosus* (Meinert, 1872), widely distributed in China, but it can easily be distinguished by antennae usually 20+20 articles, ocelli 21–27, usually 25 on each side, arranged in five irregular rows, with the posterior ocellus the largest, Tömösváry’s organ slightly smaller than the adjacent ocelli; commonly 9+9 coxosternal teeth, porodonts between the fourth and fifth or the fifth and sixth outer teeth; the posterior angles of TT 4, 6, 7, 9, 11 and 13 with obvious triangular projections; coxal pore formula 13–25, irregularly arranged, legs 1–14 with anterior and posterior accessory spurs, lacking posterior accessory spurs of legs 15. DaC spine on legs 12–15. Female gonopods with 4+4 moderately small coniform spurs, arranged in two rows. The type specimens are deposited in the collection of the Institute of Myriapodology, School of Life Sciences, Hengshui University, Hengshui, P. R. China.

## Introduction

Wood (1862) established the genus *Bothropolys*, based on three North American species. Of them, Crabill (1955) chose *Bothropolysnobilis* Wood, 1862 as the type species, the typification being confirmed by Jeekel (1963). Subsequent scholars found that *B.nobilis* was a junior synonym of *Lithobiusmultidentatus* Newport, 1844 (Eason 1972). Currently, the "A World Catalogue of Centipedes (Chilopoda)" (Bonato et al. 2016) has described 31 species; furthermore, with three additional Chinese (*Bothropolysedgecombei* Ma, Song & Zhu, 2009; *Bothropolysstoevi* Ma, Song and Zhu, 2009; *Bothropolysjiuensis* Qin, Qiao, Huang, Lin, Su & Zhang, 2017) entries that remain unlisted in the official catalogue, the total number reaches 34 species within the genus *Bothropolys*, mostly known from North America and East Asia; mostly in temperate forests, both coniferous and broadleaved, from low altitude sites up to 2300 m; sometimes in caves, but specialised species are unknown (Zapparoli and Edgecombe 2011). *Bothropolys* is characterised by the following combination of characters: body length 15–40 mm. Antenna with 20 articles or thereabouts. Ocelli 1+10 – 1+40. Forcipular coxosternal teeth usually 5+5 to 11+11. Tergites with or without posterior triangular projections. Female gonopods with tridentate claw, 2+2 – 4+4 spurs (Zapparoli and Edgecombe 2011).

For nearly half a century, only Park et al. (2016) conducted mitochondrial DNA research on unnamed species of *Bothropolys*; Ganske et al. (2020) studied the sequence fragments of 18S rRNA, 28S rRNA, 16S rRNA and COI for four species of *Bothropolys*. Scholars Wang and Mauriès (1996) reviewed and listed four species of *Bothropolys* in China, Ma et al. (2008a) reviewed and listed seven species of *Bothropolys* in China, Ma et al. (2008b) have described and published two new species, Ma (2012) documented the two described species within the Hengshui Lake National Nature Reserve, Ma et al. (2014) reviewed and listed nine species of *Bothropolys* in China, Qin et al. (2017) have described and published one new species, bringing the number of *Bothropolys* species known from China to a total of eleven. It is therefore evident that the taxonomy of the genus *Bothropolys* is relatively poorly advanced both domestically and internationally, with most studies focusing on external morphological descriptions and seldom addressing molecular sequence data, especially in molecular phylogenetic analyses. The present study deals with the description and illustration of a new species of the genus *Bothropolys* from the Yintiaoling National Nature Reserve in Wuxi County, Chongqing Municipality, south-western China.

## Materials and methods

Specimens were collected by hand under leaf litter or stones and preserved in 75% ethanol. Illustrations and measurements were produced using a ZEISS SteREO Discovery V20 microscope equipped with an Abbe drawing tube and an ocular micrometer and Axiocam 512 colour. The colour description is based on specimens fixed in 75% ethanol. The body length is measured from the anterior margin of the cephalic plate to the posterior end of the postpedal tergite. The terminology of the external anatomy follows Bonato et al. (2010). The type specimens examined are deposited in the Institute of Myriapodology, School of Life Sciences, Hengshui University, Hengshui, China (HUSLSIM).

## Taxon treatments

### Bothropolys biserialis

Gu, Pei & Ma sp. nov.

3D31AA4F-B039-5E39-83AD-F01A143BE205

D2F39BA1-344C-41DA-AFE8-C8DA9DD1FBC4

#### Materials

- **Type status:** Holotype. **Occurrence:** recordedBy: Ma Hui-Qin; individualCount: 1; sex: female; lifeStage: adult; occurrenceID: FD5CD38E-3352-545B-827D-88A60185501B; **Taxon:** scientificName: Bothropolysbiserialis sp. nov.; kingdom: Animal; phylum: Arthropoda; class: Chilopoda; order: Lithobiomorpha; family: Ethopolyidae; genus: Bothropolys; **Location:** country: P. R. China; stateProvince: Chongqing; locality: Red flag management and protection station, Yintiaoling National Nature Reserve, Wuxi County, Chongqing Municipality, Southwest China; verbatimElevation: 2169 m; verbatimCoordinates: 31°32′14.95′′N, 109°41′53.65′′E
- **Type status:** Paratype. **Occurrence:** recordedBy: Ma Hui-Qin; individualCount: 2; sex: 1 male, 1 female; lifeStage: adult; occurrenceID: 0C87EAEB-6750-540A-9A0F-3506DFEED232; **Taxon:** scientificName: Bothropolysbiserialis sp. nov.; kingdom: Animal; phylum: Arthropoda; class: Chilopoda; order: Lithobiomorpha; family: Ethopolyidae; genus: Bothropolys; **Location:** country: P. R. China; stateProvince: Chongqing; locality: Red flag management and protection station, Yintiaoling National Nature Reserve, Wuxi County, Chongqing Municipality, Southwest China; verbatimElevation: 2169 m; verbatimCoordinates: 31°32′14.95′′N, 109°41′53.65′′E
- **Type status:** Other material. **Occurrence:** recordedBy: Ma Hui-Qin; individualCount: 1; sex: male; lifeStage: adult; occurrenceID: 2906A727-DC89-50FD-90D6-9A39595A7DB0; **Taxon:** scientificName: Bothropolysbiserialis sp. nov.; kingdom: Animal; phylum: Arthropoda; class: Chilopoda; order: Lithobiomorpha; family: Ethopolyidae; genus: Bothropolys; **Location:** country: P. R. China; stateProvince: Chongqing; locality: Guanshan management and protection station, Yintiaoling National Nature Reserve, Wuxi County, Chongqing Municipality, Southwest China; verbatimElevation: 1844 m; verbatimCoordinates: 31°28′25.45′′N, 109°47′6.97′′E

#### Description

**Figure 1.**
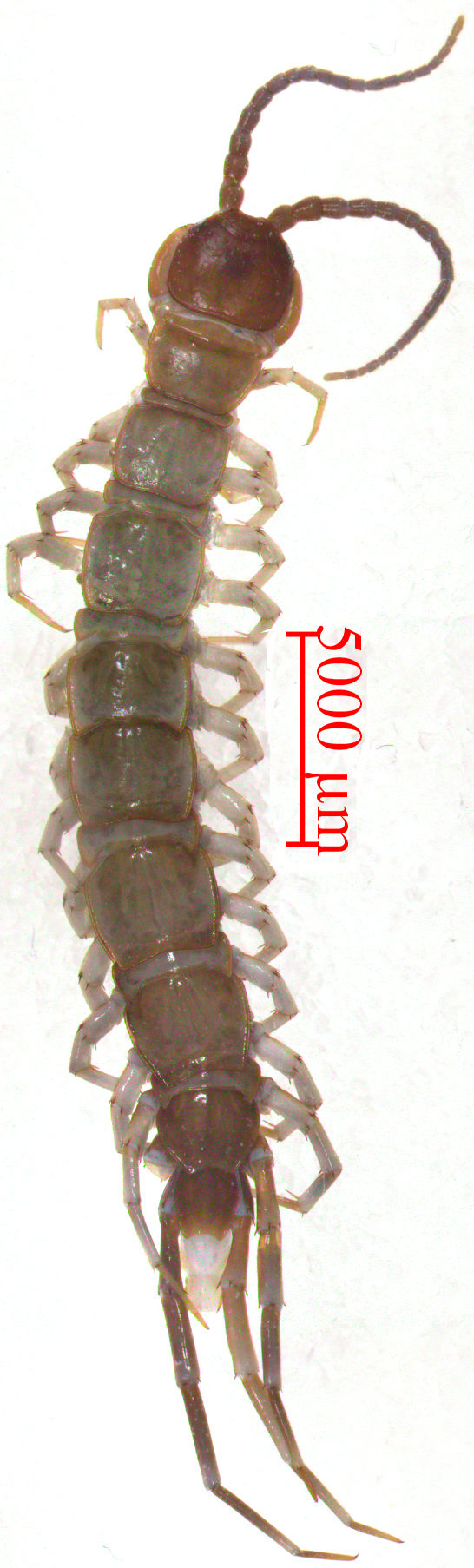
*Bothropolysbiserialis* sp. nov., Female holotype, habitus, dorsal view.

**Body** (Fig. 1). Body 21.96–25.05 mm long, cephalic plate 2.00–2.92 mm long, 2.23–2.93 mm wide; holotype body 25.05 mm long, cephalic plate 2.56 mm long, 2.96 mm wide.

**Colour**. The antennae brown to greyish-brown and then to yellow, tergites dark brown to light brown or greyish-brown and then to dark brown, TT 1, 2, 14 and 15 darker. The cephalic plate dark brown, pleural region grey-white, sternites pale brown to dark brown. The distal part of forcipules dark brown, basal and proximal parts of forcipules, forcipular coxosternite grey-brown and SS 14 and 15 dark yellow-brown. All the legs gradually turn from grey to yellow, with the distal part of the tarsi being a darker colour.

**Antennae**. Some 19–20 articles, usually 20+20 articles, holotype 19+20 articles. The first antennal article is approximately equal in length and width, the remaining articles obviously longer than wide, the distalmost articles still significantly longer than wide, 2.6–3.0 times as long as wide (Fig. 1); abundant setae on the antennal surface, fewer on the basal articles, gradual increasing in density to approximately the seventh article, then more or less constant.

**Figure 2.**
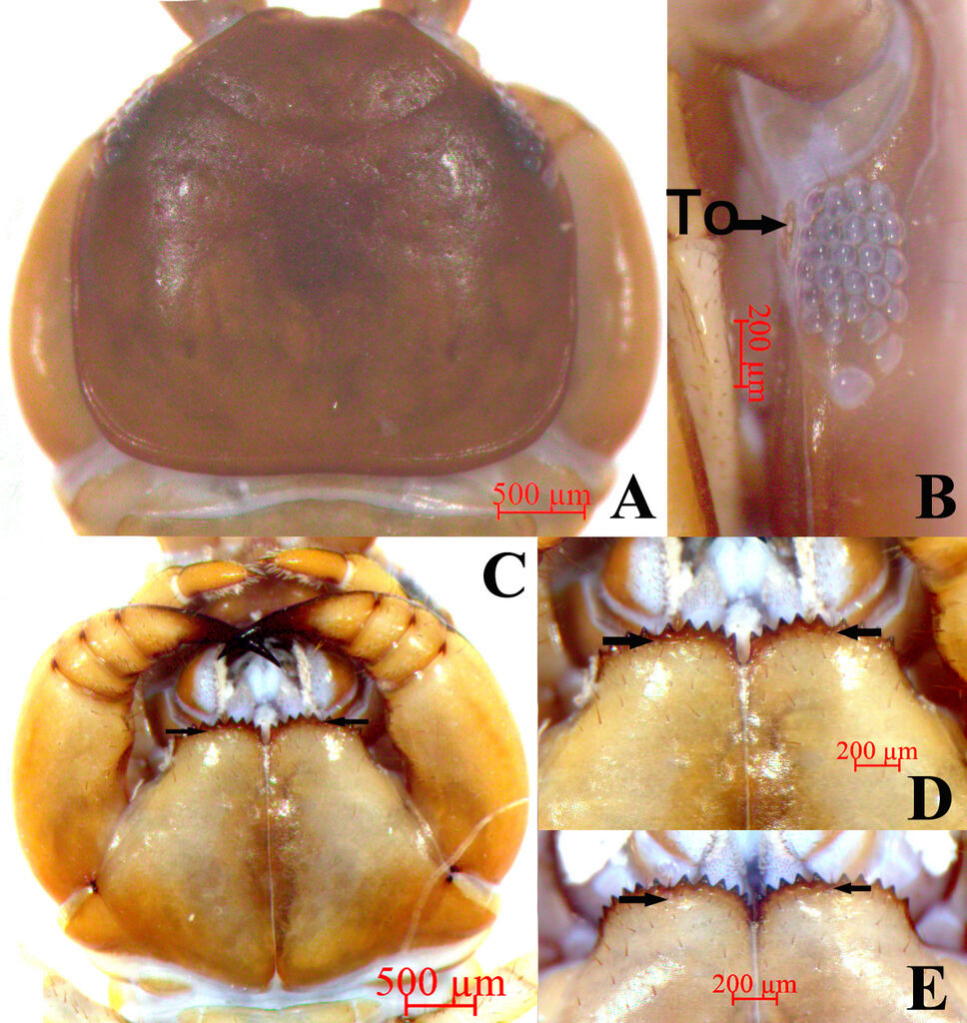
*Bothropolysbiserialis* sp. nov., Female holotype (A-D), other material (E). **A** cephalic plate, dorsal view; **B** ocelli and Tömösváry’s organ (To), lateral view; **C-D** forcipular coxosternite, ventral view; **E** forcipular coxosternite, ventral view;

**Cephalic plate**. Smooth, convex, approximately equal in length and width; tiny setae emerging from pores scattered very sparsely over the whole surface and the whole surface of cephalic plate is covered with a fine hexagonal mesh; frontal marginal ridge with shallow anterior median furrow; short to long setae scattered along the marginal ridge of the cephalic plate. The lateral marginal ridge discontinuous, posterior margin continuous, almost straight, evidently wider than lateral marginal ridge, especially in the middle (Fig. 2A).

**Ocelli**. Some 21–27, commonly 25, holotype ocelli 27, oval to rounded ocelli on each side, arranged in five irregular rows, the posterior ocellus the largest. Ventral ocelli closely arranged than the dorsal, domed, translucent and usually dark purple pigmented (Fig. 2B).

**Tömösváry’s organ** (Fig. 2B). Close to the ocelli, situated at anterolateral margin of the cephalic plate, the surrounding sclerotised area narrow, slightly smaller than the adjoining ocelli.

**Coxosternite**. Subtrapezoidal (Fig. 2C), anterior margin wide, lateral margins approximately equal to medial margins; median diastema moderately deep, a slightly wider U shape; the whole surface of coxosternite is covered with a fine hexagonal mesh, anterior margin with 8–11 acute triangular teeth, commonly 9+9, holotype 9+9; porodonts slightly thicker, almost transparent, between the fourth and fifth outer teeth, one specimen between the fifth and sixth outer teeth with slight bulge at base (Fig. 2C–E); long scattered setae on the ventral side of coxosternite, longer setae near the dental margin.

**Figure 3.**
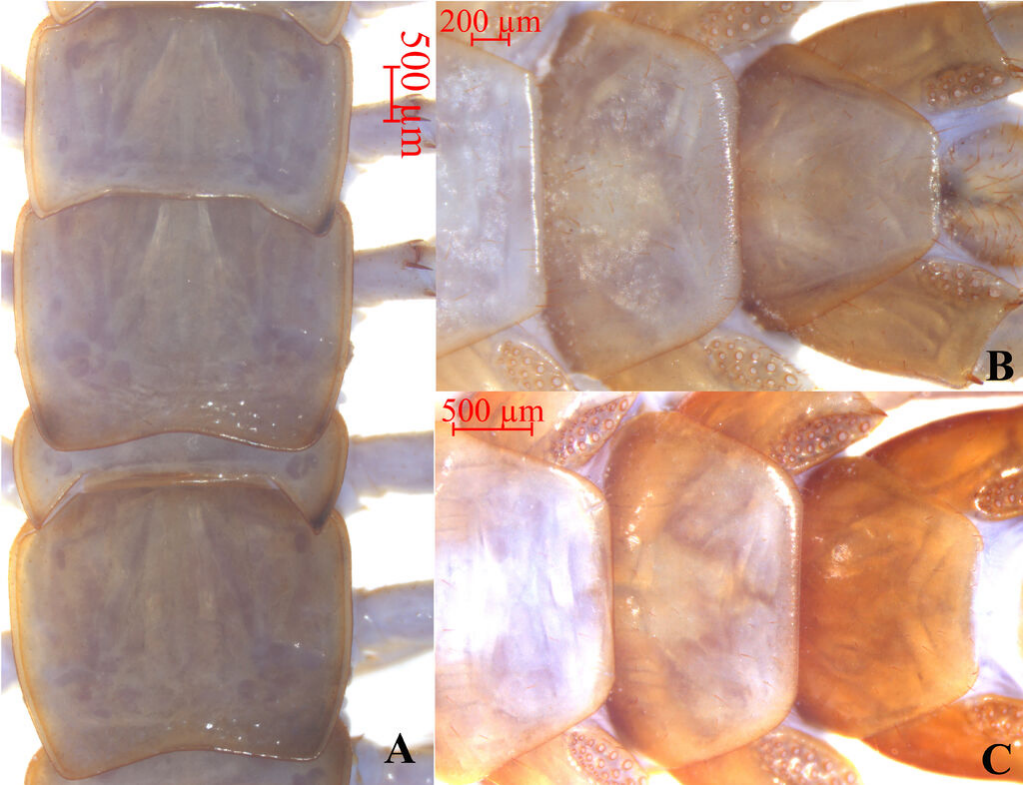
*Bothropolysbiserialis* sp. nov., other male (A), other female (B) and Female holotype (C). **A** TT 7–10; **B** SS 14–15; **C** SS 14–15.

**Tergites**. With wrinkles, dorsum slightly convex; short to long tiny setae emerging from pores scattered sparsely over the entire surface, near the margin with few long setae. Posterior margin T1 almost straight, posterior margin of TT 3 slightly concave, posterior margin of TT 5, 7, 8, 10, 12 and 14 concave (Fig. 1 and Fig. 3A). The lateral marginal ridges of all tergites continuous, posterior marginal ridge of TT 1 and 3 continuous, posterior marginal ridge of TT 5, 8, 10, 12 and 14 discontinuous. The posterior angles of TT 4, 6 and 7 with obvious triangular projections, the posterior angles of TT 9, 11 and 13 with acute triangular projections, TT 8 and 10 are nearly equal, widest. Posterior angles of all tergites rounded, without triangular projections. From short and thick, but miniscule setae scattered very sparsely over the surface, with more setae at both the anterior and the posterior corners, especially in anterior corners and they are mostly short.

**Sternites**. Posterior side of sternites narrower than anterior, generally inverted trapezoidal, smooth; setae emerging from sparsely scattered pores on the surface and lateral margin, very few short setae scattered sparsely amongst them, with more setae at both the anterior and the posterior corners. In male, only S 14 has extremely dense, short and coarse setae, especially in the latter part (Fig. 3B); in females, the setae on SS 14 and 15 are denser compared to the others (Fig. 3C).

**Legs**. Relative robust, tarsi well-defined on legs 1–15. All legs with moderately long curved claws; legs 1–14 with anterior and posterior accessory spurs, anterior accessory spurs moderately thinner and shorter, forming a moderately small angle with the claw, posterior accessory spurs slightly longer and more robust, forming a comparatively large angle with the claw; lacking posterior accessory spurs of legs 15. From short to long setae very sparsely scattered over the surface of coxa, trochanter, prefemur, femur and tibia of legs 1–13; more setae on the tarsal surfaces, especially in the ventral, setae on the dorsal and ventral surfaces slightly longer than the anterior and posterior, some obvious thicker setae arranged in one row on the ventral surfaces of tarsi of legs 1–13; with setae significantly uniform and homogeneous on legs 14 and 15, having dense glandular pores on the inside from femur to tibia of legs 14 and 15. Legs 14 and 15 longer and thicker than the anterior legs in both of the female and male, male legs 15 moderately thicker and stronger than those of the female. Ta2 6.4–10.6 times longer than wide, Ta2 57.0%–64.3% length of Ta1 on legs 15 in female; Ta2 5.9–11.1 times longer than wide, Ta2 54.0%–68.6% length of Ta1 on legs 15 in male. Leg plectrotaxy given in Table 1 and Table 2.

**Coxal pores**. Slightly oval or round, 13–25 irregularly arranged, the number of coxal pores in legs 15 is slightly less than that in the front, with more than 13 to 20, 18/17–22/20–25/23–18/17 in holotype. Coxal pore-field set in a relatively shallow groove, its fringe with a slight prominence and moderately long setae sparsely scattered over the surface.

**Figure 4.**
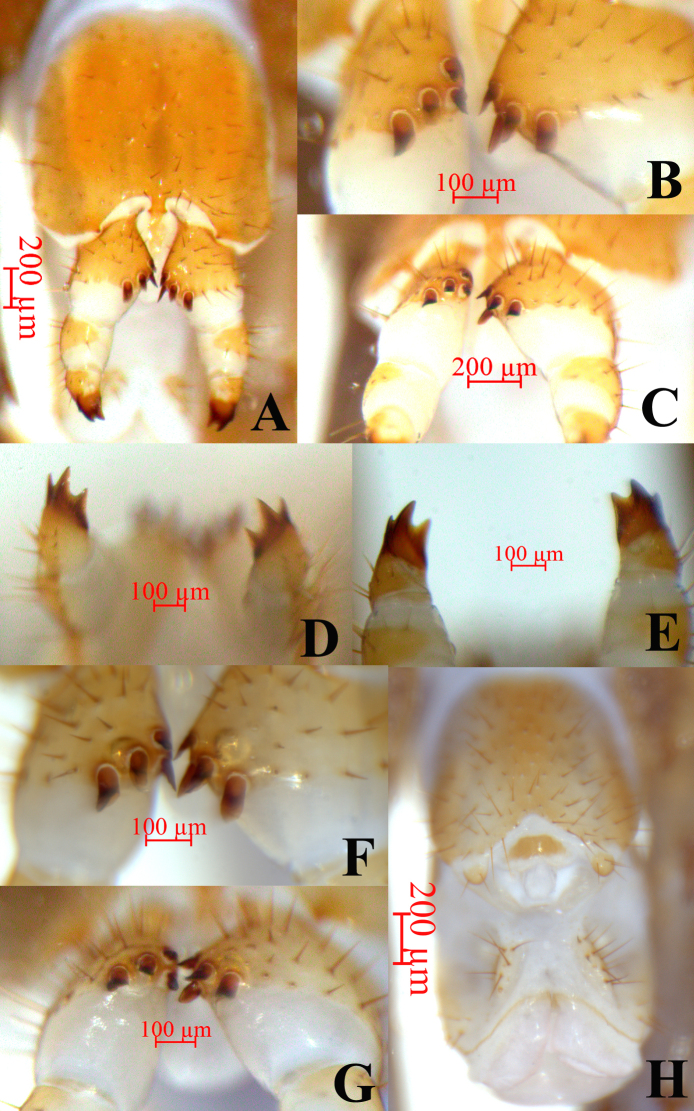
*Bothropolysbiserialis* sp. nov., Female holotype (A–E) and other female (F–G) and paratype male (H). **A** posterior segments and gonopods, ventral view; **B** spurs of the first article of gonopods arranged in two rows, ventral view; **C** spurs of the first article of gonopods arranged in two rows, posterior view; **D** tridentate apical claw of gonopods, dorsal view; **E** tridentate apical claw of gonopods, ventral view; **F** tridentate apical claw of gonopods, dorsal view; **G** tridentate apical claw of gonopods, ventral view; **H** posterior segments and gonopods.

**Female**. S 15 anterior margin broader than posterior, generally an inverted trapezoid, posterior angles rounded, postero-medially straight. Moderately long to short setae relatively densely scattered on S 15 surface. Surface of the lateral sternal margin of genital segment well chitinised, posterior margin of genital sternite deeply concave between condyles of gonopods, except for a small, median tongue-shaped bulge. Relatively long setae very sparsely scattered over ventral surface of the genital segment, slightly more setae on posterior part, especially along the posterior edge. Gonopods: first article fairly broad, bearing 23–25 moderately long setae, arranged in four irregular rows, with 4+4 small coniform spurs, inner two spurs slightly smaller than the outer, arranged in two rows (Fig. 4 A–C and F–G); second article with 9–10 long setae in the ventral, arranged in three irregular rows; third article with 4–5 long setae in the ventral, arranged in two irregular rows, with a tridentate apical claw (Fig. 4D–E).

**Male**. S 15 posterior margin narrower than anterior, postero-medially straight, generally an inverted trapezoid, sparsely covered with long to short setae, the setae on the edges being longer; sternite of genital segment evidently smaller than the female, usually sclerotised; posterior margin deeply concave between the gonopods, without medial bulge. Short to long setae equally scattered on the ventral surface of the genital segment. Gonopods short and wide, flat, with 2–4 long setae, apically slightly sclerotised (Fig. 4H).

**Habitat**. The specimens here studied were collected under the deciduous leaves of pine trees around the mountain road.

#### Diagnosis

In accordance with the grouping of species proposed in the genus *Bothropolys*, the new species differs from other congeners in having the antennae composed of 19–20, commonly 20+20 articles, ocelli 21–27, usually 25 on each side, arranged in five irregular rows, with the posterior ocellus the largest, Tömösváry’s organ slightly smaller than the adjacent ocelli; commonly 9+9 coxosternal teeth, porodonts lying between the fourth and fifth outer teeth; the posterior angles of TT 4, 6, 7, 9, 11 and 13 with obvious triangular projections; coxal pore formula 13–20, irregularly arranged. Female gonopods with 3+3 or 4+4 moderately small coniform spurs, apical claw of the third article tridentate.

#### Etymology

The specific name meaning is "two rows", and is intended to emphasise that the spurs of the first article of female gonopods are arranged in two rows.

#### Notes

**Figure 5.**
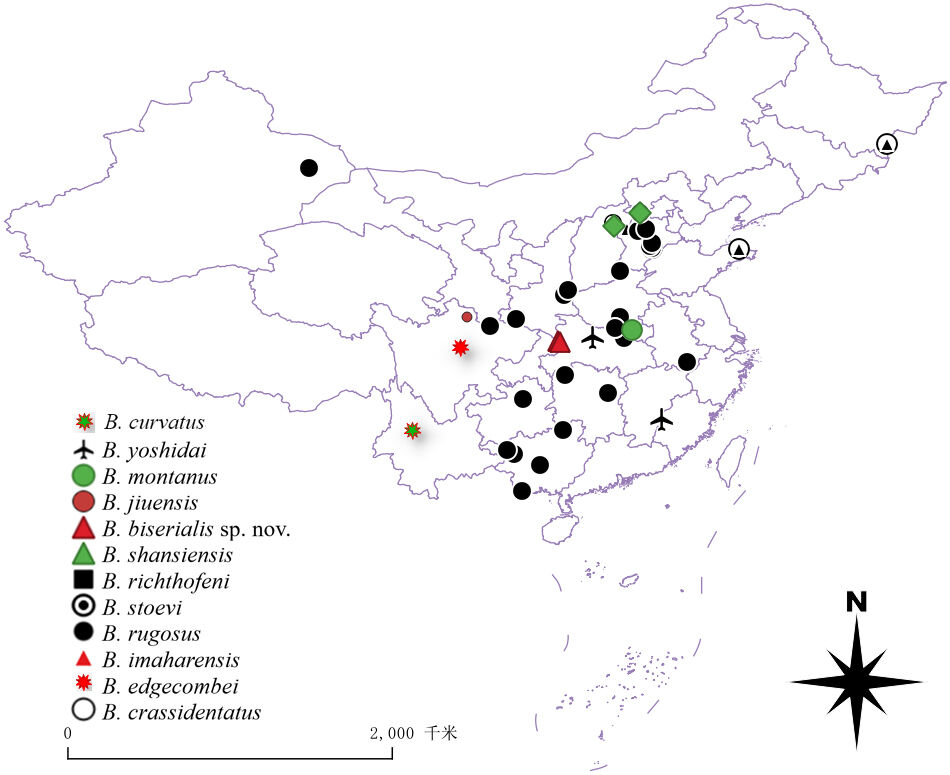
The collecting localities map of Chinese records species of *Bothropolys* Wood, 1862.

To assist in the identification of the Chinese species belonging to the genus *Bothropolys*, an identification key is offered, emphasising characters that can be examined without high-magnification microscopy; moreover, these characters are specific to the taxa occurring in China. A map with the collection localities of the 12 Chinese species of the genus *Bothropolys* Wood, 1862 is also presented (Fig. 5).

## Identification Keys

### Key to the Chinese species of the genus *Bothropolys* Wood, 1862.

**Table:** 

1	The posterior angles of T 2 with triangular projections	*B.edgecombei* (Ma, Song and Zhu, 2009)
–	The posterior angles of T 2 rounded	2
2	The posterior angles of T 4 rounded	3
–	The posterior angles of T 4 with triangular projections	8
3	Without porodonts in coxosternite anterior margin, 35 ocelli	*B.montanus* (Verhoeff, 1938)
–	Porodonts present in coxosternite anterior margin, no more than 30 ocelli	4
4	Porodonts between the third and fourth outer teeth	*B.richthofeni* (Verhoeff, 1938)
–	Porodonts lying posterolateral to lateralmost tooth	5
5	The posterior angles of TT 6 and 7 rounded	*B.stoevi* (Ma, Song and Zhu, 2009)
–	The posterior angles of TT 6 and 7 with triangular projections	6
6	Female gonopods having 3+3–5+5 spurs	*B.rugosus* (Meinert, 1872)
–	Female gonopods having 2+2 spurs	7
7	Legs 1–15 with anterior and posterior accessory spurs, DaC spine on legs 9–15	*B.imaharensis* (Verhoeff, 1937)
–	legs 14–15 lacking accessory spurs, no DaC spine on legs 9–15	*B.crassidentatus* (Takakuwa, 1949)
8	Porodonts between the fourth and fifth or the fifth and sixth outer teeth	9
–	Porodonts are not between the fourth and fifth or the fifth and sixth outer teeth	10
9	Ocelli arranged in 5 irregular rows, lacking posterior accessory spurs of legs 15	*B.biserialis* sp. nov.
–	Ocelli arranged in 4 irregular rows, lacking accessory spurs of legs 15	*B.curvatus* (Takakuwa, 1939)
10	Porodonts between the third and fourth outer teeth	*B.yoshidai* (Takakuwa, 1939)
–	Porodonts are not between the third and fourth outer teeth	11
11	Porodonts between the second and third outer teeth, no DaC spine on legs 9–10	*B.shansiensis* (Takakuwa, 1949)
–	Porodonts between the first and second outer teeth, DaC spine on legs 9–10	*B.jiuensis* (Qin, Qiao, Huang, Lin, Su & Zhang, 2017)

## Discussion

Morphologically, the new species seems to be especially similar to *B.yoshidai* Takakuwa, 1939 (Takakuwa 1939) from Hubei and Fujian Provinces, China, as well as Japan and North Korea, with which it shares antennae 19–20 articles; usually 20+20 articles; 20–26 ocelli on each side; 8–11 prosternal teeth; the posterior angles of T 4, 6, 7, 9, 11 and 13 with obvious triangular projections; female gonopods having 4+4 spurs; legs 1–14 with anterior and posterior accessory spurs. However, the new species can be distinguished from *B.yoshidai* easily by the following characters: prodonts between the fourth and fifth or the fifth and sixth outer teeth vs. porodonts between the three and four outer teeth in *B* . *yoshidai*; the posterior ocellus the largest vs. the posterior two ocelli the largest in *B.yoshidai*; only lacking posterior accessory spurs of legs vs. lacking accessory spurs of legs 15 in *B.yoshidai*; DaC spine present on legs 12–15 vs. DaC spine present on legs 11–15 in *B.yoshidai*.

In morphological characters, the new species seems also to be very similar to *B.curvatus* Takakuwa, 1939 (Takakuwa 1939), from the Yunnan Province, China, as well as Japan, with which it shares the antennae with 20+20 articles, porodonts between the fourth and fifth or the fifth and sixth outer teeth and the posterior angles of TT 4, 6, 7, 9, 11 and 13 are with triangular projections and legs 1–14 with anterior and posterior accessory spurs. However, the new species can easily be distinguished from *B.curvatus* by the following characters: the ocelli arranged in commonly five irregular rows, vs. the ocelli arranged in commonly four irregular rows; T 4 with obviously triangular projections, vs. T 4 with feeble projections in *B.curvatus*; only lacking posterior accessory spurs of legs vs. lacking accessory spurs of legs 15 in *B.curvatus*; no DaC spine on legs 11, vs. DaC spine on legs 11 in *B.curvatus*.

In addition, the new species also seems to be quite similar to *B.rugosus* (Meinert, 1872) (Ma et al. 2008a), a form widely distributed in China, with which it shares the antennae with 20+20 articles, 8–11 prosternal teeth, the posterior angles of TT 9, 11 and 13 with obvious triangular projections; female gonopods having 4+4 spurs; legs 1–13 with anterior and posterior accessory spurs. However, the new species can readily be distinguished from *B.rugosus* by the following characters: Tömösváry’s organ smaller than the adjoining ocelli, vs. larger than the adjoining ocelli in *B.rugosus*; porodonts between the fourth and fifth or the fifth and sixth outer teeth vs. porodonts lying posterolateral and adjacent to the lateralmost tooth in *B.rugosus*, posterior angles of TT 4, 6 with obviously triangular projections, vs. posterior angles of T 4 rounded, posterior angles of T 6 feebly triangular in *B.rugosus*; anterior and posterior accessory spurs of legs 14 present, anterior accessory spurs of legs 15 present vs. accessory spurs of legs 14 and 15 lacking in *B.rugosus*.

## Supplementary Material

XML Treatment for Bothropolys biserialis

## Figures and Tables

**Table 1.** Leg plectrotaxy of *Bothropolysbiserialis* sp. nov., females.

legs	dorsal					ventral				
	C	Tr	P	F	Ti	C	Tr	P	F	Ti
1			amp	ap	a			mp	amp	am
2-9			amp	ap	ap			mp	amp	am
10-11			amp	ap	ap			amp	amp	am
12-13	a		amp	p	p		m	amp	amp	am
14	a		amp	p	p	a	m	amp	amp	a
15	a		amp	p		am	m	amp	am	a

**Table 2.** Leg plectrotaxy of *Bothropolysbiserialis* sp. nov., males.

Legs	dorsal					ventral				
	C	Tr	P	F	Ti	C	Tr	P	F	Ti
1			amp	a	a			mp	amp	am
2-3			amp	ap	a			mp	amp	am
4-9			amp	ap	ap			mp	amp	am
10-11			amp	ap	ap			amp	amp	am
12			amp	p	p		m	amp	amp	am
13	a		amp	p	p	a	m	amp	amp	am
14	a		amp	p	p	a	m	amp	amp	a
15	a		amp	p		a	m	amp	amp	a

## References

[B12568542] Bonato Lucio, Edgecombe Gregory, Lewis John, Minelli Alessandro, Pereira Luis, Shelley Rowland, Zapparoli Marzio (2010). A common terminology for the external anatomy of centipedes (Chilopoda). ZooKeys.

[B12568516] Bonato L, Chagas Junior A, Edgecombe GD, Lewis JGE, Minelli A, Pereira LA, Shelley RM, Stoev P, Zapparoli M A World Catalogue of Centipedes (Chilopoda). http://chilobase.biologia.unipd.it..

[B12568573] Crabill R. E.Jr (1955). Concerning the genotypes of *Bothropolys*, *Polybothrus* and *Eupolybothrus* (Chilopoda: Lithobiomorpha: Lithobiidae). Entomological News.

[B12666507] Eason E. H. (Edward Holt) (1972). The type specimens and identity of the species described in the genus *Lithobius* by C. L. Koch and L. Koch from 1841 to 1878 (Chilopoda: Lithobiomorpha). Bulletin of the British Museum (Natural History), Zoology.

[B12562924] Ganske Anne‐Sarah, Vahtera Varpu, Dányi László, Edgecombe Gregory D., Akkari Nesrine (2020). Phylogeny of Lithobiidae Newport, 1844, with emphasis on the megadiverse genus *Lithobius* Leach, 1814 (Myriapoda, Chilopoda). Cladistics.

[B12568584] Jeekel C. A.W (1963). The generic and subgeneric names of the European Lithobiidae generally referred to *Polybothrus* Latzel, 1880 (Chilopoda
Lithobiida). Entomologische Berichten.

[B12568663] Ma H, Song D, Zhu M (2008). A review of the Chinese species of *Bothropolys* Wood, 1862 (Chilopoda: Lithobiomorpha: Lithobiidae). Zootaxa.

[B12568672] Ma Hui-Qin, Song Da-Xiang, Zhu Ming-Sheng (2008). Two new species of the genus *Bothropolys* Wood, 1862 (Chilopoda: Lithobiomorpha: Lithobiidae) from China. Entomologica Fennica.

[B12568470] Ma H (2012). A preliminary study on *Bothropolys* from Hengshui Lake. Journal of Hengshui University.

[B12568623] Ma HUIQIN, Pei SUJIAN, Hou XIAOJIE, Zhu TIEGANG, Wu DAYONG, Gai YONGHUA (2014). An annotated checklist of Lithobiomorpha of China. Zootaxa.

[B12568771] Park Sin Ju, Choi Eun Hwa, Hwang Jae Sam, Hwang Ui Wook (2016). The complete mitochondrial genome of a centipede *Bothropolys* sp. (Chilopoda, Lithobiomorpha, Lithobiidae). Mitochondrial DNA Part A.

[B12568711] Qin Wen, Qiao Penghai, Huang YanGan, Lin Gonghua, Su Jianping, Zhang Tongzuo (2017). A new species of *Bothropolys* and a new record of *Lithobiusmagnitergiferous* (Lithobiidae) from the Qinghai-Tibet Plateau, China. Biologia.

[B12582432] Takakuwa Y. (1939). *Bothropolys*-Arten aus Japan.. Transactions of the Natural History Society of Formosa.

[B12568898] Wang D, Mauriès J. P., Geoffroy J. - J., Mauriès J. - P., Nguyen D - J M (1996). Acta Myriapodologica. Mémoires du Muséum National d'Histoire Naturelle.

[B12571073] Wood H. C. (1862). On the Chilopoda of North America, with a catalogue of all the specimens in the collection of the Smithsonian Institution. Journal of the Academy of Natural Sciences of Philadelphia.

[B12571502] Zapparoli M, Edgecombe G. D., A Minelli (2011). Treatise on Zoology - Anatomy, Taxonomy, Biology, The Myriapoda.

